# Comparative Epidemiology of Rabbit Haemorrhagic Disease Virus Strains from Viral Sequence Data

**DOI:** 10.3390/v15010021

**Published:** 2022-12-21

**Authors:** Carlo Pacioni, Robyn N. Hall, Tanja Strive, David S. L. Ramsey, Mandev S. Gill, Timothy G. Vaughan

**Affiliations:** 1 Department of Environment, Land, Water and Planning, Arthur Rylah Institute for Environmental Research, Heidelberg, VIC 3084, Australia; 2 Environmental and Conservation Sciences, Murdoch University, Perth, WA 6150, Australia; 3 Commonwealth Scientific and Industrial Research Organisation, Health and Biosecurity, Canberra, ACT 2601, Australia; 4 Department of Statistics, University of Georgia, Athens, GA 30602, USA; 5 Institute of Bioinformatics, University of Georgia, Athens, GA 30602, USA; 6 Department of Biosystems Science and Engineering, Eidgenössische Technische Hochschule Zurich, 4058 Basel, Switzerland

**Keywords:** calicivirus, RHDV, phylodynamics, RNA virus, coalescent, Birth Death models

## Abstract

Since their introduction in 1859, European rabbits (*Oryctolagus cuniculus*) have had a devastating impact on agricultural production and biodiversity in Australia, with competition and land degradation by rabbits being one of the key threats to agricultural and biodiversity values in Australia. Biocontrol agents, with the most important being the rabbit haemorrhagic disease virus 1 (RHDV1), constitute the most important landscape-scale control strategies for rabbits in Australia. Monitoring field strain dynamics is complex and labour-intensive. Here, using phylodynamic models to analyse the available RHDV molecular data, we aimed to: investigate the epidemiology of various strains, use molecular data to date the emergence of new variants and evaluate whether different strains are outcompeting one another. We determined that the two main pathogenic lagoviruses variants in Australia (RHDV1 and RHDV2) have had similar dynamics since their release, although over different timeframes (substantially shorter for RHDV2). We also found a strong geographic difference in their activities and evidence of overall competition between the two viruses.

## 1. Introduction

Since their introduction in 1859, European rabbits (*Oryctolagus cuniculus*) have had a devastating impact on agricultural production and biodiversity in Australia, with competition and land degradation by rabbits listed as key threatening processes under the *Commonwealth’s Environmental Protection and Biodiversity Act (1999)*. 

Biocontrol agents, with the most important being the rabbit haemorrhagic disease virus 1 (RHDV1), constitute the most important landscape-scale control strategies for rabbits in Australia. However, the presence of several co-occurring strains in Australia substantially increases the complexity of their possible interactions in the field.

RHDV1, classified as genotype GI.1 following Le Pendu, et al. [1] (from now on referred to as RHDV1), escaped from Wardang Island, South Australia, in October 1995, where it was being tested as a biological control agent. The virus became established nationwide within 12 months [2]. Following an initial rabbit population reduction of more than 90% [3,4], ongoing monitoring revealed that the efficacy of RHDV1 was highly variable throughout Australia, with average rabbit population abundance reductions of 67% in lower rainfall areas compared to 28% in high rainfall areas [5,6,7]. Seasonality of disease outbreaks also became evident, where, within a site, the disease typically ceased to be detected in late summer [8].

At some locations, serological analyses detected antibodies cross-reacting to RHDV1 in samples collected prior to the arrival of RHDV1, suggesting that RHDV1 effectiveness was likely impeded by the presence of a related, non-pathogenic calicivirus [6,9,10], which was later identified as rabbit calicivirus (RCV) Australia-1 [11,12,13]. According to the nomenclature proposed by Le Pendu, et al. [1], Australian RCV viruses are classified as genotype GI.4, and can be further subdivided into GI.4a, GI.4b, and GI.4c variants.

In 2015, a virus previously only detected in Europe (RHDV2), was detected in Australia [14], and successive retrospective serological tests detected evidence of its presence dating back to March 2014 [15]. Phylogenetic analyses revealed this virus to be a recombinant RHDV2 comprising non-structural sequences of RHDV1 and structural sequences of RHDV2 (i.e., genotype GI.1bP-GI.2). 

RHDV2 rapidly spread across Australia within 18 months of its arrival, becoming the dominant circulating pathogenic lagovirus [16]. Four other pathogenic lagoviruses are also known to be present in Australia: RHDVa-K5, a GI.1a virus released nationally as a new biocontrol tool in 2017 [17]; RHDVa-Aus, a recombinant GI.4eP-GI.1a virus detected in eastern Australia in 2014 but not deliberately released [18]; GI.4cP-GI.2, a recombinant variant that arose in Australia through recombination between RCV and RHDV2 [19]; and GI.4eP-GI.2, an additional recombinant variant that arose in Australia through recombination between RHDVa-Aus and RHDV2 [20]. Thus, currently, seven distinct lagovirus variants are present in Australia: (1) RHDV1 genotype GI.1; (2) RCV genotype GI.4; (3) RHDV2 genotype GI.1bP-GI.2; (4) RHDVa-K5 genotype GI.1a; (5) RHDVa-Aus genotype GI.4eP-GI.1a; (6) RHDV2 genotype GI.4cP-GI.2; (7) RHDV2 genotype GI.4eP-GI.2. 

In our previous analysis of a limited number of RHDV1 sequences (n = 68) spanning from 1995 to 2014, (which only included the gene that encodes the capsid protein VP60), we demonstrated that phylodynamic analyses can be greatly informative for the investigation of epidemiological parameters of RHDV1 in the field [21]. However, we also identified limitations in these analyses as the sample size, and geographical and temporal distribution of sampling were limited. Furthermore, we pointed out that while general trends can be detected in coalescent-based analyses that do not take into account the geographical heterogeneity of RHDV1, biases can be introduced when analysing these data without explicitly taking into account geographical structuring. 

Here, we expand the original RHDV1 dataset to include (mostly) whole-genome sequences, increasing the sample size and the geographical and temporal distribution of samples of this genotype and including in our analyses a large dataset of RHDV2 whole-genome sequences and all available RCV sequences. Using phylodynamic models including a structured Birth-Death model, [22,23,24,25] to analyse the available RHDV molecular data, we aimed to investigate the epidemiology of various strains (with a focus on RHDV1 and RHDV2) and evaluate whether the molecular data are supportive of early reports that RHDV2 originated in Australia in NSW/ACT in 2014, as suggested by serological and molecular detection of this virus [15]. Finally, we attempt to gain insight into possible competitive dynamics between RHDV1 and RHDV2 strains. 

## 2. Methods

Whole-genome sequences for Australian lagoviruses were generated in previous studies [16,18,19].

We used the whole-genome sequences for the three major lagoviruses circulating in Australia. The dataset includes 51 RHDV1, 185 RHDV2, and 44 RCV-A1 sequences. We also included in the alignment 95 RHDV1 VP60 sequences (by padding missing data with Ns) in order to maximise sample size (total n = 146) and the time and space represented in the data for this genotype. We selected only GI.1, GI.4, and GI.1bP-GI.2 sequences in the final dataset, excluding GI.1a, G1.4eP-GI.1a, GI.4cP-GI.2, and GI.4eP-GI.2 (i.e., we did not include any recombinants). That was performed because the analyses we conducted (see below) do not model recombination. Note that we refer to RHDV1 and RHDV2 as genotypes or strains, interchangeably. The sampling span was different for each virus: the data included samples between 1995 and 2017 for RHDV1 (Figure S1), between 2015 and 2018 for RHDV2 (Figure S2), and between 2007 and 2014 for RCV (Figure S3). The time of sampling was expressed as fractional years. For those sequences for which the exact date of collection was unknown (accession number: GU373617, GU373618, EU650679, EU650680), we used the midpoint of the possible range as the season of collection (Table S1).

The analyses that we use here apply either a coalescent or Birth-Death model to exploit the information accumulated in the genetic data of the viruses to infer the demographic changes over time or estimate parameters relevant to epidemiological studies. By applying a molecular clock, time can be expressed in calendar units rather than the number of substitutions, allowing for a better appreciation of the viruses’ dynamics and, as the evolutionary processes are inferred, it is possible to describe events that were not directly observed. All analyses were conducted in BEAST 2.6.1 [26], except for the Skygrid analysis (see below), which was conducted in BEAST 1.10 [27]. The times of population size changes used in these analyses were set as summarised in Table 1. We used a lognormal relaxed molecular clock [28] because it was demonstrated to be the most suitable clock model for RHDV1 [21]. In all analyses, we used bModelTest [29] to take into account the uncertainty in the substitution model, except for the Skygrid analysis (see below) because this model is implemented in BEAST 1.10 [29] where bModelTest is not available. For this analysis, we used a GTR + Γ + I substitution model. We used a gamma prior (shape = 1, scale = 0.001) for the clock rate, which encompasses the expected range for an RNA virus [30]. 

**Figure 1:**
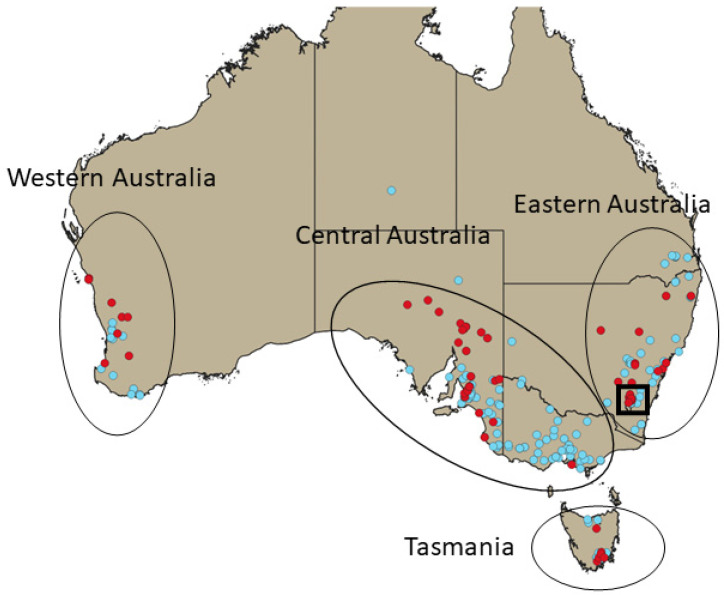
Map showing the sampling locations for RHDV1 (red) and RHDV2 (cyan) sequences and the four regions these were classified into. Thick square indicates samples collected in proximity to the Australian Capital Territory (ACT).

We analysed the data with the Bayesian skyline plot BSP, [22] and Skygrid [24] coalescent tree priors. We also used a structured sampler to account for (spatial) structure in our data. This was achieved by separating the data into four regions (Central Australia, Eastern Australia, Western Australia, and Tasmania, Figure 1) and analysing them using the multitype Birth-Death model (aka Birth-Death with Migration Model, BDMM) [25] as implemented in the BEAST package BDMM-prime (https://github.com/tgvaughan/BDMM-Prime, accessed on 24 June 2022). Structured analyses were limited to the RHDV1 and RHDV2 datasets because, based on our previous experience with this model [21], it was judged that the sample size of the RCV-A1 was too limited for such analyses. For BDMM analyses, we used a lognormal prior for *R*_e_ (log mean = 0.5 and log standard deviation = 1) and the total removal rate *δ* (δ=μ+ψr, where µ is the death rate, ψ is the sampling rate and r is the removal probability) [23] (log mean = 2 and log standard deviation = 2). We used a uniform distribution bounded between 0 and 1 for the sampling proportion parameter but fixed this parameter to zero prior to the earliest sample collection. We also fixed the removal probability parameter to zero because it is unlikely that our sampling regime affected lagovirus outbreaks [21]. Because of the previously noted inverse correlation between *R*_e_ and δ [21], we used a common δ across all epochs while leaving *R*_e_ to vary. For RHDV1, we used a lognormal prior around the origin parameter (log mean = 3.1 and log standard deviation = 0.1), as Australia was pathogenic lagoviruses free before its release. For RHDV2, we also used a lognormal prior distribution for the origin parameter (log mean = −1.39 and log standard deviation = 0.5 with an offset of 3.7). 

To test the hypothesis that the pathogenic lagovirus genotypes are outcompeting each other, we conducted a follow-up Skygrid analysis, taking advantage of the extension of this model that allows the inclusion of a covariate [31]. Under this framework, the population history timeline is divided up into different time intervals and the virus’s effective population size is assumed to be piecewise constant, assuming different values during each interval. The virus population size for the k-th interval is modelled as:

Ln(PopSize_k_) = *βX*_k_ + w_k_ (1)

where *β* is a regression coefficient (which could be interpreted as the effect size for the predictor), *X*_k_ is the covariate value corresponding to the k-th time interval, and w_k_ is an intercept term modelled as a Gaussian Markov random field to impose temporal dependence. If *β* > 0 the analysis demonstrates that the virus population size has a positive association with the predictor, on the contrary, *β* < 0 would indicate a negative association. We, therefore, repeated the RHDV2 analysis using RHDV1 median population size estimates obtained from the analysis without covariates as follows:

ln(PopSize_RHDV2,k_) = *β* ln(PopSize_RHDV1,k_) + w_k_

We decided to analyse the RHDV2 data using estimated RHDV1 median population size as a covariate (as opposed to analysing RHDV1 data using estimated RHDV2 median population size as a covariate) in order to minimize the time frame for which the covariate value is unobserved. In particular, while RHDV1 population size estimates are available for most of the RHDV2 population history, most of the RHDV1 population history corresponds to pre-RHDV2 years. 

Using a similar approach, we also re-implemented the BDMM analysis co-analysing both genotypes concurrently, but this time we used a joint prior for the estimation of the respective reproductive numbers. For this prior, we used a multivariate lognormal distribution and estimated the covariance (which itself had a normal prior with mean = 0 and standard deviation = 10) with the expectation that, if the two strains are under competitive exclusion, this would be negative, without intersecting zero. In this analysis, we fixed the RHDV2 reproductive number to 0.01 in epochs prior to its arrival in Australia.

Tracer [32] was used to ensure convergence of the two MCMC that were run for each model (that is, the independent analyses sampled the same parameter space), and to generate the data for the BSP and the skyride plot. Two runs were then combined for final analysis. Skyline plots were generated with the R package BEASThandleR (https://github.com/carlopacioni/BEASTHandleR, accessed on 24 June 2022). All input files are available from the corresponding author.

## 3. Results

### 3.1. BSP Analyses

**Figure 2:**
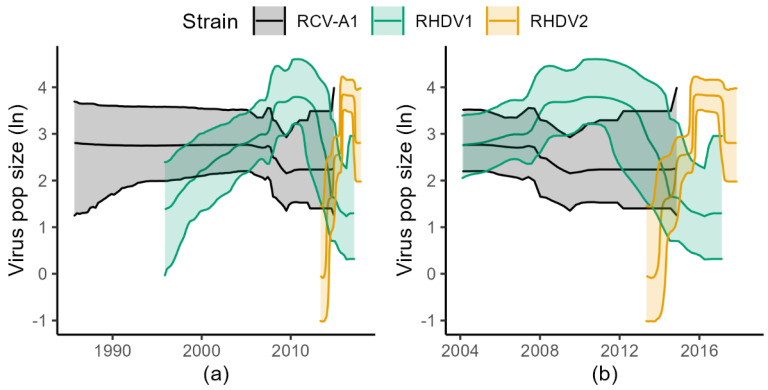
Bayesian Skyline Plots of the three lagoviruses. The left panel (**a**) shows the complete timeline (until the root of RCV-A1, in the early 1980s). In the right panel (**b**) the *x*-axis is limited to the time interval between 2004 and 2018 to facilitate comparison of the dynamics of the three viruses. Shaded areas are the 95% Highest Probability Density and solid lines are the posterior medians (in natural log).

**Figure 4:**
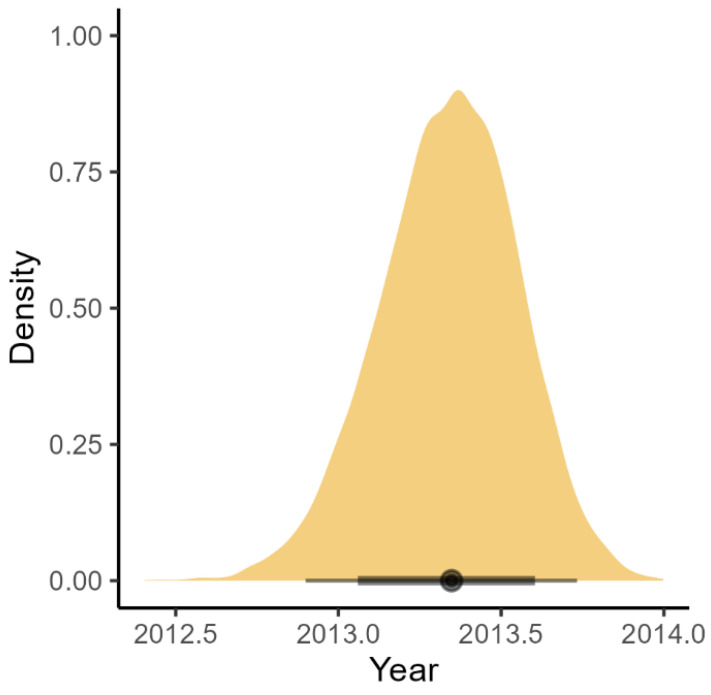
Density plot of the estimated age of the root of the RHDV2 phylogenetic tree based on the Bayesian Skyline Plot analysis. The dot on the black bar shows the posterior median, the thick bar the 80%, and the thin bar the 95% credible intervals.

The BSP analysis of RCV-A1 indicated that the population size of this virus remained relatively stable during the last 30 years (Figure 2). The RHDV1 strain showed an increase in population size until 2009, followed by a plateau until 2013 when the population size commenced a decline which has continued until the time of the most recent sampling (Figure 2). Although the date of release of RHDV1 is known with certainty [2 October 1995], it is interesting to note that our analysis estimated the correct age of the root of the tree, with an error of only three months (March 1995, with the 95% CrI upper limit estimated to be July 1995, Figure 3). Analysis of RHDV2 showed a strong increasing trend subsequent to its arrival (which is estimated to have occurred in the autumn of 2013 (Figure 4)) until 2016 when the virus plateaued and then sharply declined towards the end of the Australian summer of 2017 (late 2017). The Highest Probability Density (HPD) intervals of the population size estimates from the RHDV2 analysis were generally narrower compared to the other two strains (except those estimates close to the root of the tree) and the analysis suggests a seasonality of the disease, as demonstrated by the regular plateau in Figure 2. In fact, with the exception of the summer of 2016, generally, there was no indication of an increase in the virus population size in the three winter months, while it consistently increased in the warmer months. This seasonal pattern was evident from soon after the virus’ arrival, as the analysis suggests that while present during winter 2013, the population size did not start increasing until spring 2013.

Between 2004 and 2010 there seems to be an inverse relationship between RHDV1 and RCV population size, where the former increases and the latter decreases, although this pattern does not continue after 2012. RHDV1 started to decline in 2012. Between 2012 and 2013, there also seems to be an inverse relationship between RHDV2 and RHDV1.

### 3.2. Skygrid Analyses

**Figure 5:**
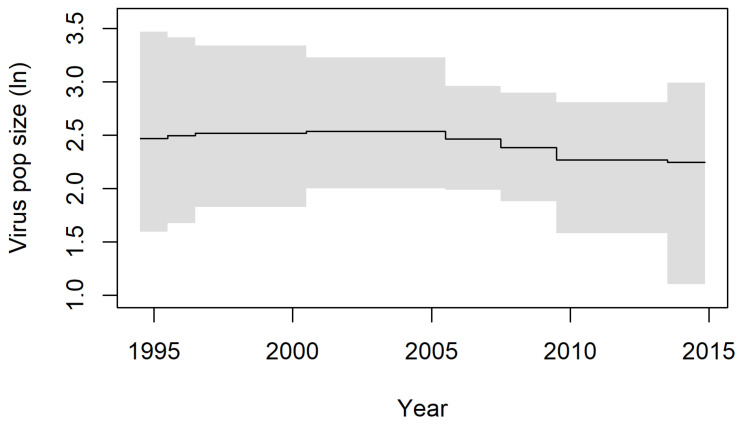
Skygrid analysis of RCV-A1. Details on the epochs used in the analysis are in Table 1. Shaded areas show the 95% Highest Probability Density and solid lines are the medians.

**Figure 6:**
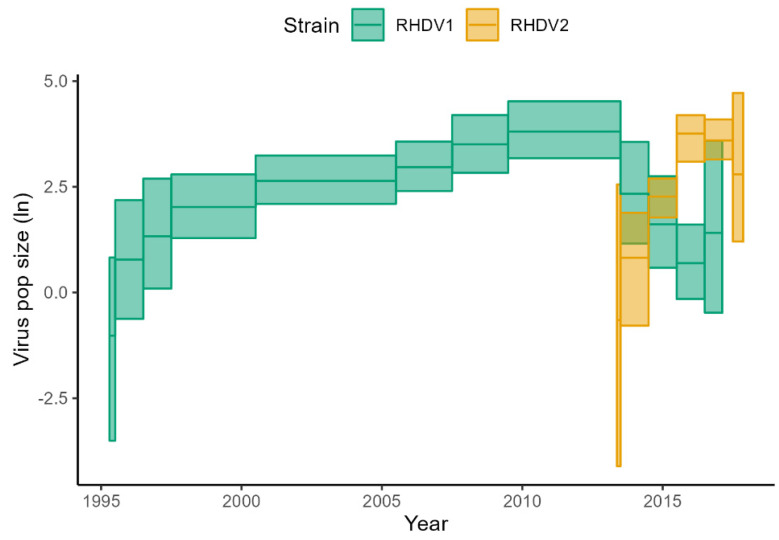
Skygrid plot of the RHDV1 and RHDV2 strains. Details on the epochs used in the analysis are in Table 1. Shaded areas show the 95% Highest Posterior Density regions and solid lines are the posterior medians.

Analyses with the Skygrid model were comparable with previous results. RCV-A1 analysis indicated that the population size of this virus remained relatively stable during the last 20 years (Figure 5). RHDV1 population size showed an increasing trend until late 2009 when it plateaued and declined from 2012 onwards (Figure 6). RHDV2 population size estimates were also consistent with BSP analysis (Figure 2). These analyses also seem to suggest an inverse relationship between the population sizes of RHDV2 and RHDV1.

### 3.3. BDMM-Prime

**Figure 7:**
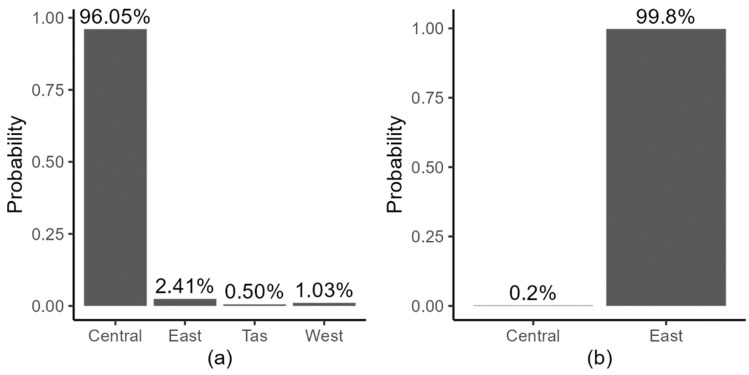
Posterior probability of the root location for RHDV1 (**a**) and RHDV2 (**b**) across the possible demes considered by the analysis. Note that for RHDV2, the Western region and Tasmania were not included in the credible intervals.

We grouped samples into four demes (Figure 1, Figures S5 and S6). The analyses assigned the location of the root for both RHDV1 and RHDV2 genotypes with very high confidence, confirming the known release site for RHDV1 and, for RHDV2, perfectly in line with rabbit ecological and serological data that indicated a rabbit demographic decline due to RHDV2 starting from the Eastern region (Figure 7).

**Figure 8:**
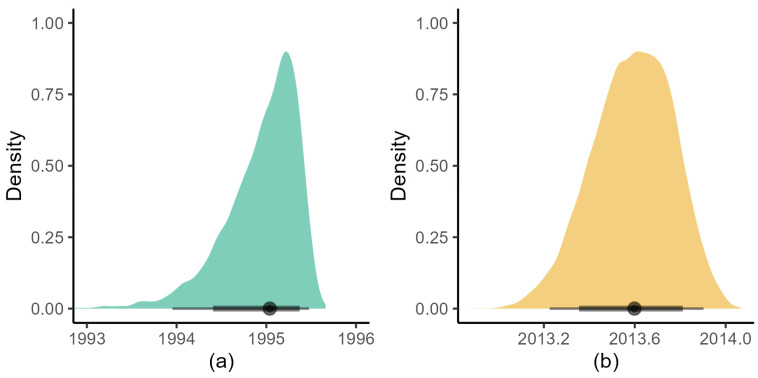
Density plot of the estimated age of the root of the RHDV1 (**a**) and RHDV2 (**b**) phylogenetic tree based on the BDMM analysis. The dot on the black bar shows the posterior median, the thick bar the 80%, and the thin bar the 95% credible intervals.

The age of the root of the RHDV1 phylogenetic tree was consistent with previous analyses, while for RHDV2, it was shifted forward by three months, with a posterior median in early August 2013 (Figure 8). 

**Figure 9:**
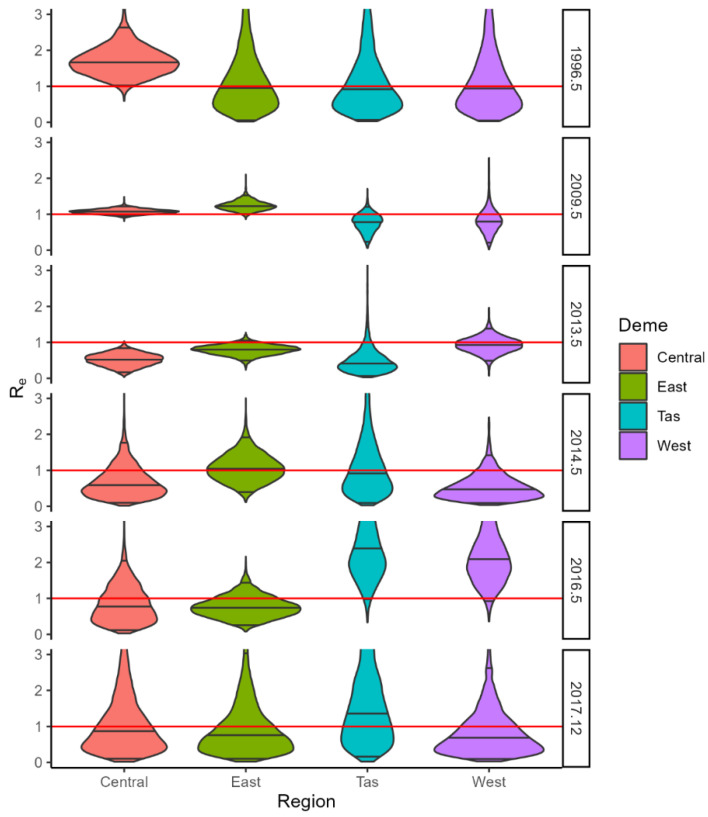
Violin plots of R_e_ densities for RHDV1 in each region (Central, Eastern, Western, and Tasmania) and epoch. Red line marks R_e_ = 1.

**Figure 10:**
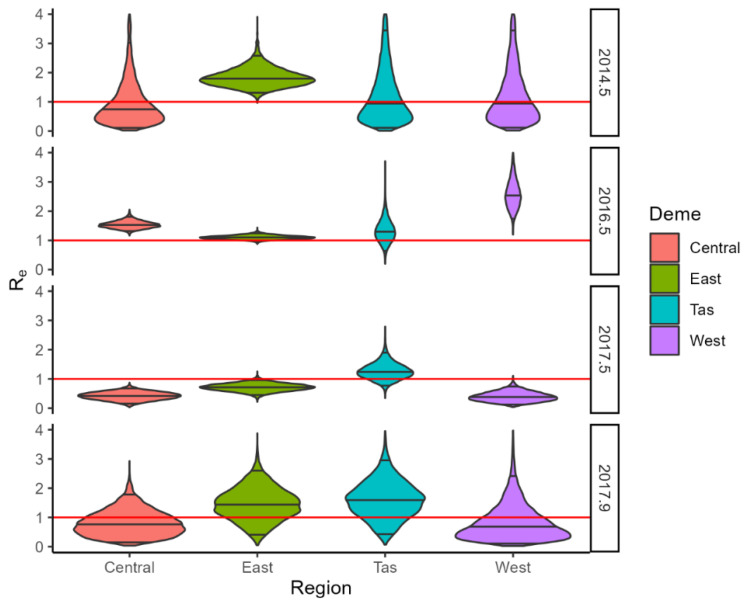
Violin plots of R_e_ densities for RHDV2 in each region (Central, Eastern, Western, and Tasmania) and epoch. Red line marks R_e_ = 1.

Interestingly, BDMM analysis clearly shows the heterogeneity in virus activity across the different regions of the landscape (Figure 9 and Figure 10). Reproductive number estimates are higher in the area of origin of the outbreaks in the first epoch (the first 6–12 months from the arrival of the virus). Then, for RHDV1, the virus activity intensified in the Eastern region between 1996 and 2009, bringing the reproductive number estimates above 1 in this region, while there were only marginal changes in R_e_ in Tasmania and Western Australia during this epoch. Between 2009 and 2013, the virus was well-established in Western Australia, with the reproductive number ~1, while it declined in Central Australia and Tasmania. The virus then maintained a reproductive number of approximately one only in the Eastern region and Tasmania in the fourth epoch (2013–2014), but a substantial increase was then evident in Tasmania and Western Australia between 2014 and 2016, while a general decline in the virus activity was evident in the final sampling epoch (2016–2017), although R_e_ still remained above 1 in Tasmania. RHDV2 moved from Eastern Australia to the Central region, Tasmania and Western Australia by the winter of 2016. However, in the Australian winter of 2017, its reproductive number remained above one only in Tasmania, before returning to a reproductive number greater than one in the Eastern region and Tasmania in late 2017.

The estimates of the infectious periods (estimated as 1/δ) for RHDV2 calculated with BDMM were about a third of those of RHDV1 (Table 2). 

### 3.4. RHDV Genotype Competitive Exclusion

**Figure 11:**
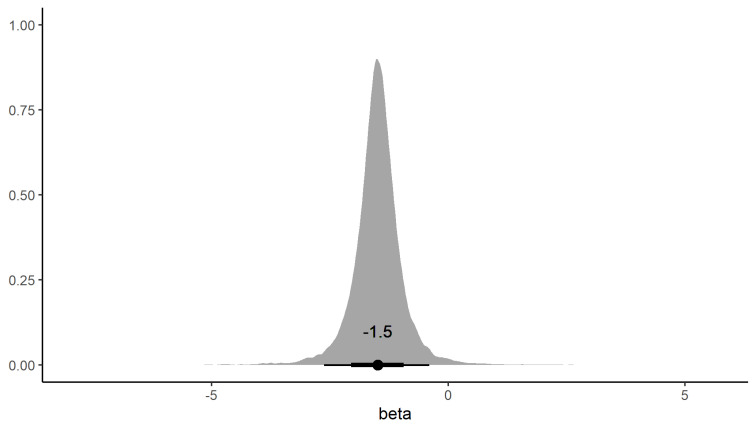
The density of the beta coefficient defining the relationship between the RHDV2 population size and RHDV1 population size (see Equation (1)). The dot on the black bar is the posterior median, the thick bar the 80%, and the thin bar the 95% credible intervals.

The Skygrid analysis of RHDV2 data using median estimated RHDV1 as a covariate returned a significant negative effect size coefficient (median = −1.5, 95% CrI = (−2.6, −0.37), Figure 11). The RHDV2 population size estimates in this analysis were also more precise than those from the analysis without using the covariate (Figure S4).

**Figure 12:**
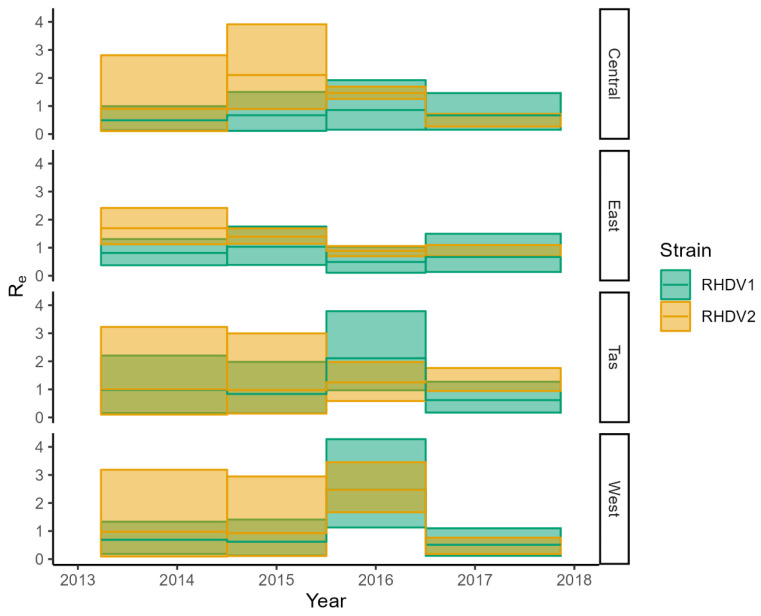
BDMM analysis of both strains (RHDV1 and RHDV2) with a joint multivariate lognormal distribution. Shaded areas show the 95% Highest Probability Density and solid lines are the posterior medians. Note how Re HPD mostly overlaps.

Contrary to the Skygrid analysis, the BDMM with a joint prior for the reproductive number did not suggest competitive exclusion between the two genotypes (median covariate estimate = 0.023, HPD = (−0.11, 0.15), Figure 12). We repeated this analysis limiting it to the two regions for which we had the largest number of sequences (i.e., Central and Eastern regions) to evaluate whether this result could be due to the limited power of the analysis. In this latter analysis, the covariance estimate was negative (posterior median = −0.057, HPD = (−0.21, 0.12)), however, the 95% HPD still encompassed zero, suggesting the difference was not significant. 

## 4. Discussion

The effort made to expand sampling beyond that provided in an earlier RHDV1 dataset [21] proved to be worthwhile because, with this more comprehensive dataset, we could fit a BDMM (which had failed previously) and gain additional insight into the spatial and temporal dynamics of the virus dynamics in the field. The results were in striking agreement with the current knowledge and understanding of lagovirus epidemiology in Australia. For example, the RHDV1 root location and age were estimated with high accuracy and the reconstructed spatial dynamics of the virus corresponded to those recorded by field monitoring [2]. For RHDV2 also, the location of arrival in Australia is in strong agreement with available serological data and field observation [14,15] and, while the molecular analysis dates RHDV2 slightly earlier than what was established with serological data [15], we think that this earlier date is plausible, given the possibility that initial, localised outbreaks of infection may have gone unnoticed. The BSP analysis suggests that RHDV2 likely arrived during the Australian winter of 2013 and stayed mostly quiescent and undetected until the summer of 2013–2014. A seasonal pattern of winter quiescence followed by outbreaks in spring was shown by our analysis. Should this earlier arrival date be correct, the delay in the RHDV2 serological and molecular detection is likely related to the fact that in the early months after its arrival, RHDV2 would have been at very low prevalence and would have been unlikely to have been detected by existing surveillance methods [14,15,16]. 

We acknowledge that the separation of the data into four demes is a simplification of the geographical compartmentalisation of the data. However, there is a trade-off between the further subdivision of the data and what can be inferred as the number of samples within each deme and epoch is reduced. Further increases in the number of demes also result in a larger number of parameters to be estimated, which increases the complexity of the model and the difficulties in obtaining accurate parameter estimates. Ideally, each Australian bioregion would be treated independently because we are aware that lagovirus activity is affected by climate, especially rainfall, but this would require very intensive sampling. We argue that our subdivision represents a reasonable compromise that allows the evaluation of broad continental dynamics while keeping the analysis feasible.

The analyses presented here also provided new insights into the dynamics of the viruses. All analyses consistently indicated that RHDV1 activity has reduced over the last five years of the study period. The RHDV2 analyses proved particularly powerful because, with a relatively large sample size, high-resolution inferences regarding population size and date of origin were possible, even given the relatively short time since the virus release in Australia. For example, the BSP analysis suggested a seasonal pattern, which has been confirmed in RHDV1 [8] and RHDV2 [33], and it was associated with rabbit breeding season and related availability of susceptible hosts [33]. Being able to use the BDMM analyses with these larger sample sizes was also a critical advantage. Not only did these analyses allow us to better appreciate the spatial dynamics of lagovirus transmission in Australia, but the estimation of the epidemiological parameters was also closer to what would have been expected given the size of the outbreaks that were observed and the consequent mortality in rabbit populations. Indeed, estimates of the reproductive parameters were well above one at times (epochs) where field data unequivocally suggested a high level of virus activity in a given region. Of great interest is also the estimation of the infectious period, as this can provide a good indication of how long the virus could be considered locally active once released for management or where it is naturally occurring. The credible interval of the RHDV1 infectious period from this study is about half the width of that inferred by Pacioni, et al. [21], where it was suggested that the sparse sampling (i.e., one sample per outbreak) hampered the estimation of this parameter at the individual level. The larger sample size of this study likely resulted in the estimation of this parameter with greater accuracy and allowed to take into account geographical compartmentalisation. It was somewhat unexpected that the infectious period of the RHDV2 (median = 3 months, CrI = 2–4 months) is of shorter duration than that of RHDV1 (median = 8.4 months, CrI = 5–13 months) as the mode of transmission is believed to be similar. However, more recent studies on Australian variants showed very rapid disease progression [34], and the findings from our study support that RHDV2 spreads rapidly through a population, depleting susceptible hosts within a short period, more quickly than RHDV1.

By comparing the RHDV1 and RHDV2 activities, there are also additional important conclusions that can be drawn. Broadly speaking, RHDV1 and RHDV2 followed a similar trajectory since their release in Australia in that both genotypes showed a dramatic initial increase in population size, which then plateaued and declined. However, our analyses suggest that the timeframe within which this occurred for RHDV2 was greatly reduced when compared with RHDV1. This observation also suggests that by now, many of the management advantages that RHDV2 offered as a rabbit biological control agent have been already exploited, as it would seem that the peak of RHDV2 activity has already passed and its activity is now in decline. Our Skygrid analysis with covariate suggests that RHDV2 may have had an overall suppressing effect on RHDV1. This result is also strongly supported by the analysis of serological data, where RHDV2 was found to negatively impact RHDV1 seropositivity [15]. This competitive exclusion seems to occur at the wider population level, as this result could not be reproduced with the concurrent BDMM analysis of the two genotypes with the estimation of the covariance. It is possible though that, given the higher complexity of the latter analysis (i.e., the large number of parameters to be estimated), the paucity of data in some regions (especially Tasmania and Western Australia) or epochs, and the resulting wider 95% HPDs, this particular analysis lacked statistical power for detecting such a suppressing effect or RHDV2 on RHDV1. We repeated this analysis only including the two regions for which we had the highest number of sequences (i.e., Central and Eastern regions). While the trend of this analysis was somewhat comparable with the result of the Skygrid analysis (the posterior median was negative), the 95% HPD still encompassed zero. We would suggest that analysis of such data with BDMM to detect competitive interactions between viruses may require substantially more intensive sampling than was available to us, and our result may largely reflect inadequate sample sizes and spatial and temporal resolution in the data. It should also be noted that subsequent to the release of RHDV1 (in 1995) until at least 2013, RHDV2 was not present in Australia. Therefore, it could not have influenced the RHDV1 activity, and possibly this may have contributed to the uncertainty in the results of this analysis. However, we cannot discount the possibility that at a local level such a competitive effect is less pronounced, while cumulative effects at a population level are evident. Indeed it is possible that the spatial dynamics of the RHDV1 and RHDV2 transmission are responsible for the persistence of both viruses as previously suggested [21]. 

## Figures and Tables

**Figure 3:**
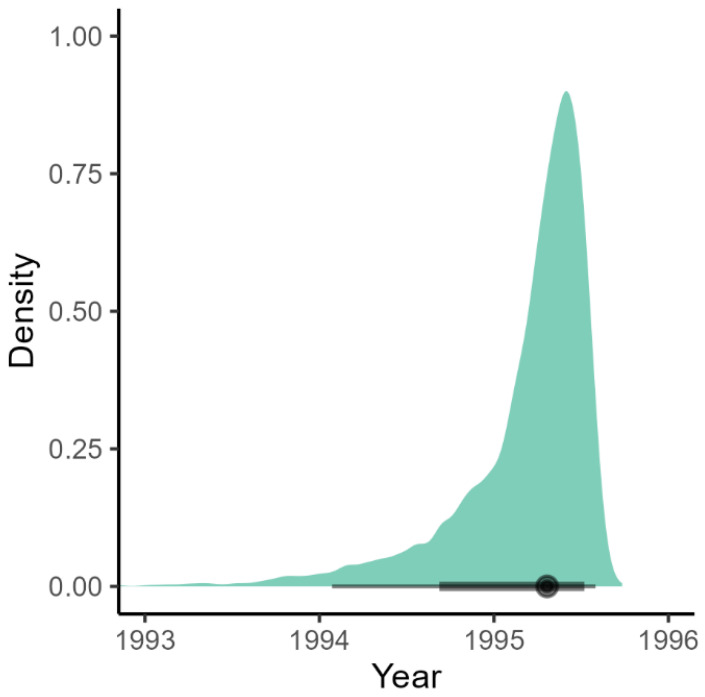
Density plot of the estimated age of the root of the RHDV1 phylogenetic tree based on the Bayesian Skyline Plot analysis. The dot on the black bar shows the posterior median, the thick bar the 80%, and the thin bar the 95% credible intervals.

**Table 1:** Summary of the number of epochs and time changes for each analysis and genotype.

Strains	Model	N Epochs	Epoch Time Changes
RHDV1	BSP	9	Free
RHDV2	BSP	5	Free
RCV-A1	BSP	5	Free
RHDV1	Skygrid	12	2017.12, 2016.5, 2015.5, 2014.5, 2013.5, 2009.5, 2007.5, 2005.5, 2000.5, 1997.5, 1996.5, 1995.5, Root
RHDV2	Skygrid	6	2017.863, 2017.5, 2016.5, 2015.5, 2014.5, 2013.5, Root
RCV-A1	Skygrid	8	2014.86, 2012.5, 2010.5, 2009.5, 2008.5, 2007.5, 2005.5, 2000.5, Root
RHDV1	BDMM	6	2017.12, 2016.5, 2014.5, 2013.23, 2009.5, 1996.5, Root
RHDV2	BDMM	4	2017.863, 2017.5, 2016.5, 2014.5, Root
RHDV1&RHDV2	BDMM	5	2017.863, 2015.5, 2014.5, 2013.23, 1996.5, Root

**Table 2:** Posterior median and 95% CrI for the estimated infectious period (estimated as 1/delta and expressed as fractional years) from BDMM-prime analyses.

Parameter	Genotype	2.75%	50%	97.50%
Infectious period	RHDV1	1.06	0.7	0.44
Infectious period	RHDV2	0.17	0.25	0.35

## Data Availability

All data used in this study are published and available in GenBank. Accession numbers are listed in the supplementary table.

## Supplementary Materials

The following supporting information can be downloaded at: https://www.mdpi.com/article/10.3390/v15010021/s1, Figure S1: Distribution of RHDV1 samples per year; Figure S2: Distribution of RHDV2 samples per year; Figure S3: Distribution of RCV-A1 samples per year; Figure S4: Skygrid analysis of RHDV2 data without covariate (red) or including the estimated median log RDHV1 population size as covariate (blue); Figure S5: BDMM RHDV1 phylogenetic tree. Branches are colour-coded by type (region), number on branches is the type probability; Figure S6: BDMM RHDV2 phylogenetic tree. Branches are colour-coded by type (region), number on branches is the type probability Table S1: Details of sequences used for this study.
