# Supplementary material for: Comparative Epidemiology of Rabbit Haemorrhagic Disease Virus Strains from Viral Sequence Data
Source: Viruses. 2022 Dec 21;15(1):21. doi: 10.3390/v15010021 (PMC9865945; doi:10.3390/v15010021)
Supplement: Supplementary file 1 [file viruses-15-00021-s001.zip › viruses-2073534-FigureS1-S6.pdf]

Supplementary figures

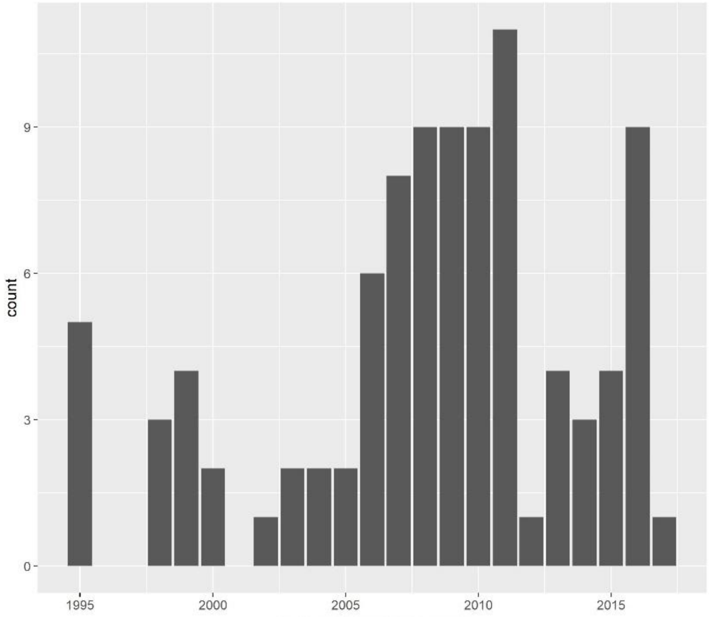

Figure S1. *Distribution of RHDV1 samples per year*

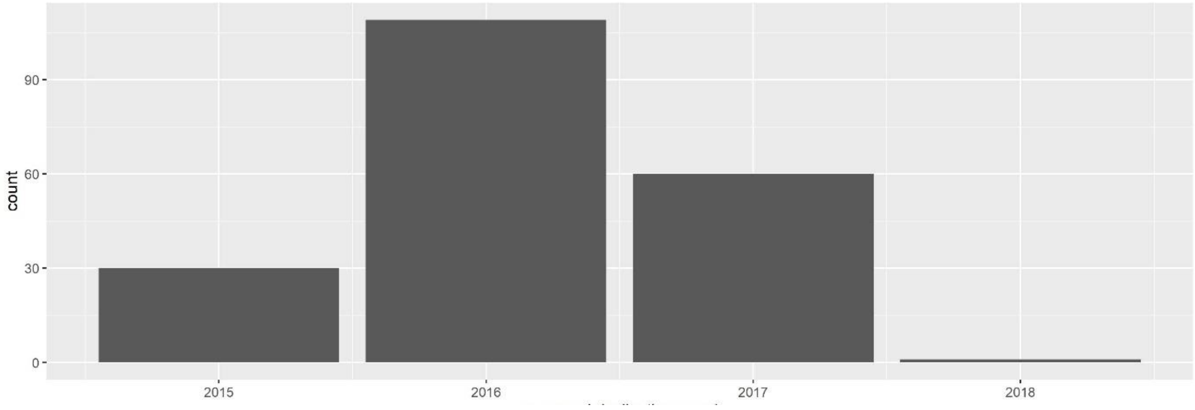

Figure S2. *Distribution of RHDV2 samples per year.*

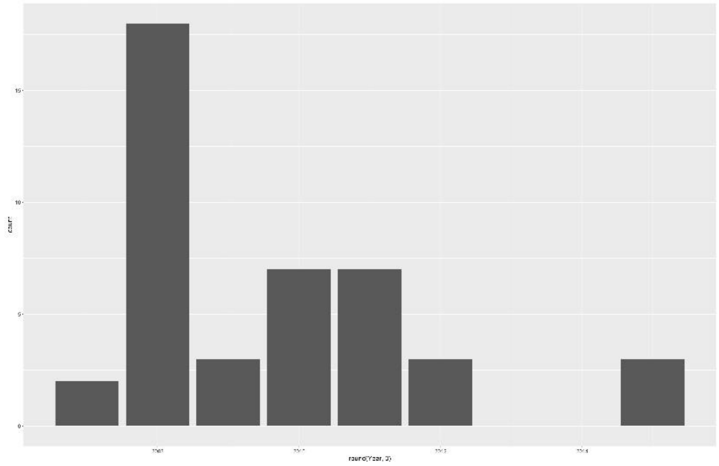

Figure S3. *Distribution of RCV-A1 samples per year.*

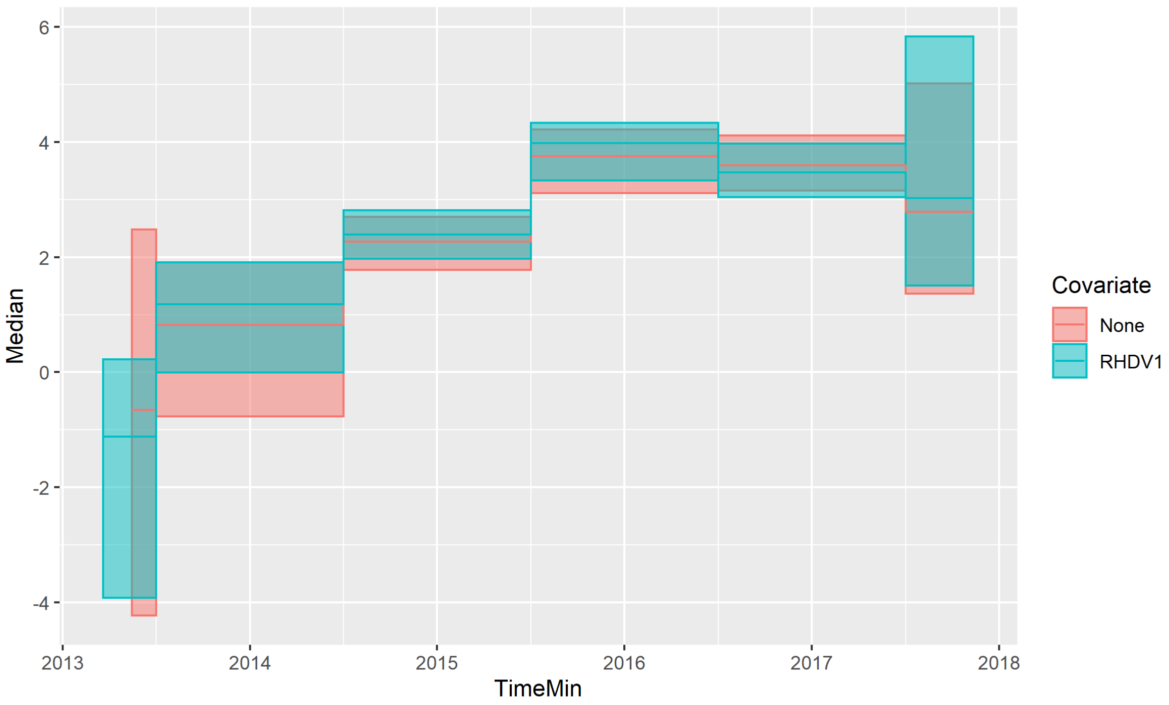

Figure S4. *Skygrid analysis of RHDV2 data without covariate (red) or including the estimated median log RDHV1 population size as covariate (blue).*

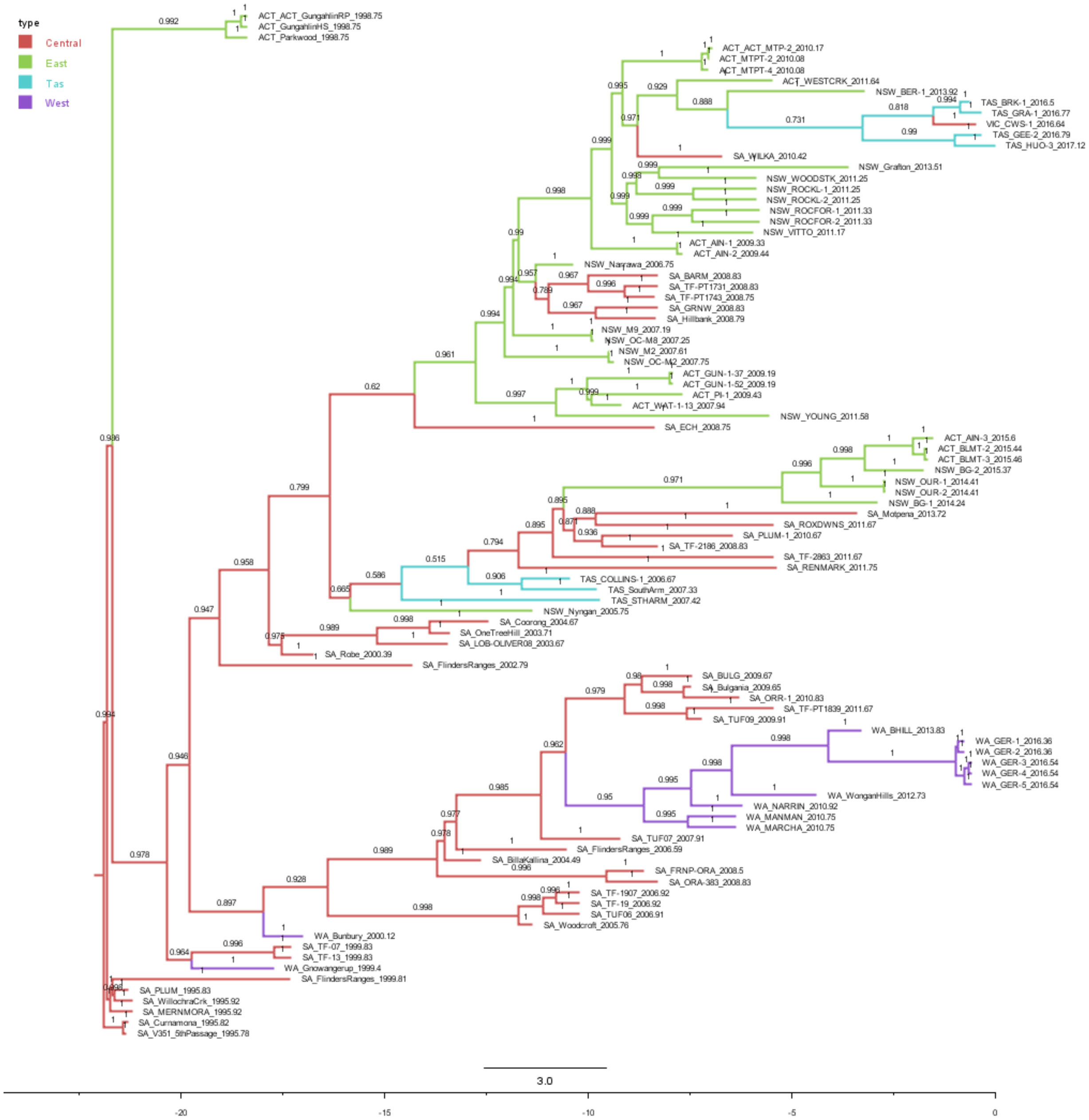

Figure S5. *BDMM RHDV1 phylogenetic tree. Branches are colour-coded by type (region), number on branches is the type probability.*

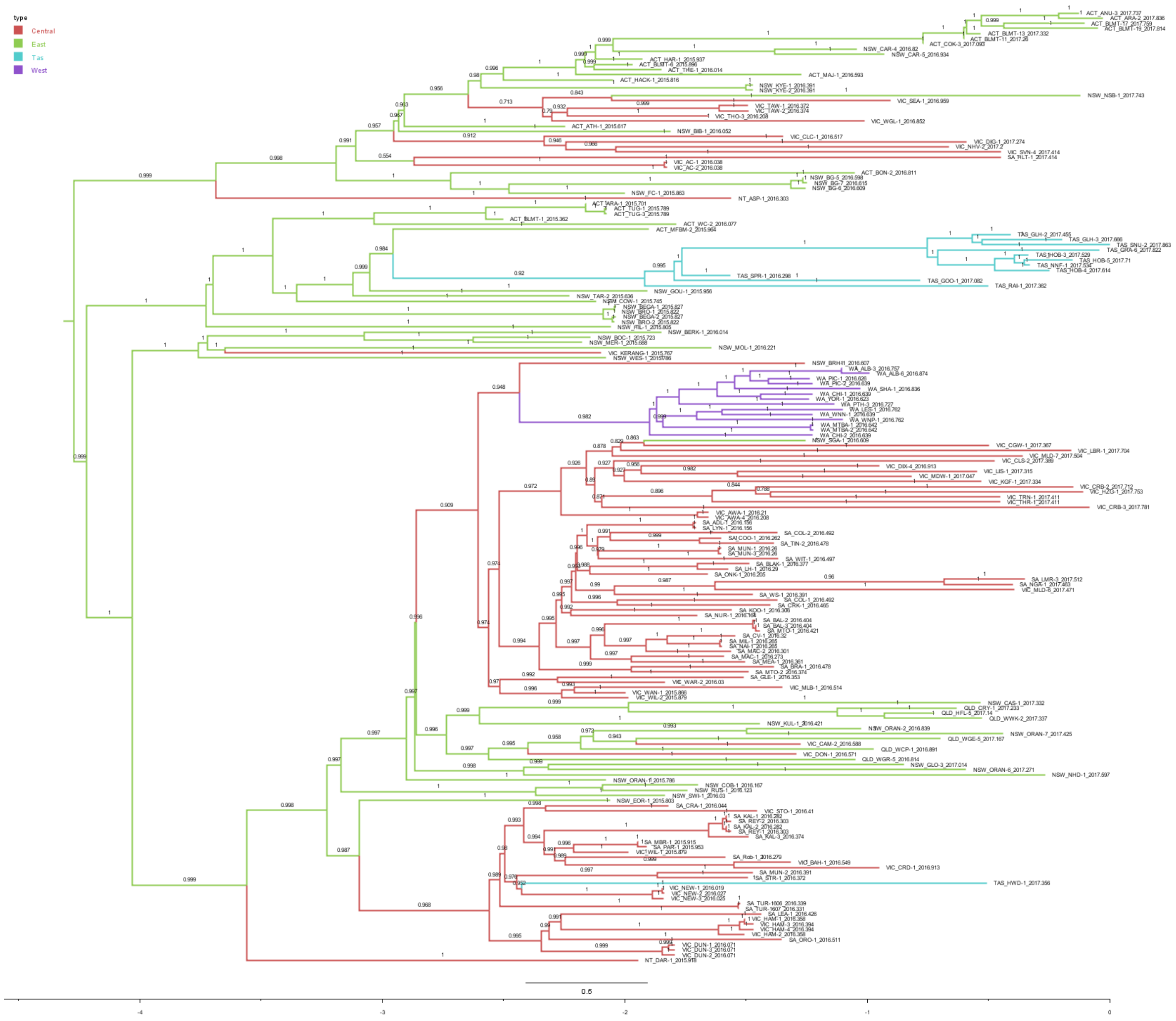

Figure S6. *BDMM RHDV2 phylogenetic tree. Branches are colour-coded by type (region), number on branches is the type probability.*
